# Experience Replay Using Transition Sequences

**DOI:** 10.3389/fnbot.2018.00032

**Published:** 2018-06-21

**Authors:** Thommen George Karimpanal, Roland Bouffanais

**Affiliations:** Engineering Product Development, Singapore University of Technology and Design, Singapore, Singapore

**Keywords:** experience replay, Q-learning, off-policy, multi-task reinforcement learning, probabilistic policy reuse

## Abstract

Experience replay is one of the most commonly used approaches to improve the sample efficiency of reinforcement learning algorithms. In this work, we propose an approach to select and replay sequences of transitions in order to accelerate the learning of a reinforcement learning agent in an off-policy setting. In addition to selecting appropriate sequences, we also artificially construct transition sequences using information gathered from previous agent-environment interactions. These sequences, when replayed, allow value function information to trickle down to larger sections of the state/state-action space, thereby making the most of the agent's experience. We demonstrate our approach on modified versions of standard reinforcement learning tasks such as the mountain car and puddle world problems and empirically show that it enables faster, and more accurate learning of value functions as compared to other forms of experience replay. Further, we briefly discuss some of the possible extensions to this work, as well as applications and situations where this approach could be particularly useful.

## 1. Introduction

Real-world artificial agents ideally need to be able to learn as much as possible from their interactions with the environment. This is especially true for mobile robots operating within the reinforcement learning (RL) framework, where the cost of acquiring information from the environment through exploration generally exceeds the computational cost of learning (Adam et al., 2012; Schaul et al., 2016; Wang et al., 2016).

Experience replay (Lin, 1992) is a technique that reuses information gathered from past experiences to improve the efficiency of learning. In order to replay stored experiences using this approach, an off-policy (Sutton and Barto, 1998; Geist and Scherrer, 2014) setting is a prerequisite. In off-policy learning, the policy that dictates the agent's control actions is referred to as the behavior policy. Other policies corresponding to the value/action-value functions of different tasks that the agent aims to learn are referred to as target policies. Off-policy algorithms utilize the agent's behavior policy to interact with the environment, while simultaneously updating the value functions associated with the target policies. These algorithms can hence be used to parallelize learning, and, thus gather as much knowledge as possible using real experiences (Sutton et al., 2011; White et al., 2012; Modayil et al., 2014). However, when the behavior and target policies differ considerably from each other, the actions executed by the behavior policy may only seldom correspond to those recommended by the target policy. This could lead to poor estimates of the corresponding value function. Such cases could arise in multi-task scenarios where multiple tasks are learned in an off-policy manner. Also, in general, in environments where desirable experiences are rare occurrences, experience replay could be employed to improve the estimates by storing and replaying transitions (state, actions, and rewards) from time to time.

Although most experience replay approaches store and reuse individual transitions, replaying sequences of transitions could offer certain advantages. For instance, if a value function update following a particular transition results in a relatively large change in the value of the corresponding state or state-action pair, this change will have a considerable influence on the bootstrapping targets of states or state-action pairs that led to this transition. Hence, the effects of this change should ideally be propagated to these states or state-action pairs. If instead of individual transitions, sequences of transitions are replayed, this propagation can be achieved in a straightforward manner. Our approach aims to improve the efficiency of learning by replaying transition sequences in this manner. The sequences are selected on the basis of the magnitudes of the temporal difference (TD) errors (Sutton and Barto, 1998), associated with them. We hypothesize that selecting sequences that contain transitions associated with higher magnitudes of TD errors allow considerable learning progress to take place. This is enabled by the propagation of the effects of these errors to the values associated with other states or state-action pairs in the transition sequence.

Replaying a larger variety of such sequences would result in a more efficient propagation of the mentioned effects to other regions in the state/state-action space. Hence, in order to aid the propagation in this manner, other sequences that could have occurred are artificially constructed by comparing the state trajectories of previously observed sequences. These virtual transition sequences are appended to the replay memory, and they help bring about learning progress in other regions of the state/state-action space when replayed.

The generated transition sequences are virtual in the sense that they may have never occurred in reality, but are constructed from sequences that have actually occurred in the past. The additional replay updates corresponding to the mentioned transition sequences supplement the regular off-policy value function updates that follow the real-world execution of actions, thereby making the most out of the agent's interactions with the environment.

## 2. Related work

The problem of learning from limited experience is not new in the field of RL (Thrun, 1992; Thomas and Brunskill, 2016). Generally, learning speed and sample efficiency are critical factors that determine the feasibility of deploying learning algorithms in the real world. Particularly for robotics applications, these factors are even more important, as exploration of the environment is typically time and energy expensive (Bakker et al., 2006; Kober et al., 2013). It is thus important for a learning agent to be able to gather as much relevant knowledge as possible from whatever exploratory actions occur.

Off-policy algorithms are well suited to this need as it enables multiple value functions to be learned together in parallel. When the behavior and target policies vary considerably from each other, importance sampling (Sutton and Barto, 1998; Rubinstein and Kroese, 2016) is commonly used in order to obtain more accurate estimates of the value functions. Importance sampling reduces the variance of the estimate by taking into account the distributions associated with the behavior and target policies, and making modifications to the off-policy update equations accordingly. However, the estimates are still unlikely to be close to their optimal values if the agent receives very little experience relevant to a particular task.

This issue is partially addressed with experience replay, in which information contained in the replay memory is used from time to time in order to update the value functions. As a result, the agent is able to learn from uncorrelated historical data, and the sample efficiency of learning is greatly improved. This approach has received a lot of attention in recent years due to its utility in deep RL applications (Adam et al., 2012; Mnih et al., 2013, 2015, 2016; de Bruin et al., 2015).

Recent works (Narasimhan et al., 2015; Schaul et al., 2016) have revealed that certain transitions are more useful than others. Schaul et al. (2016) prioritized transitions on the basis of their associated TD errors. They also briefly mentioned the possibility of replaying transitions in a sequential manner. The experience replay framework developed by Adam et al. (2012) involved some variants that replayed sequences of experiences, but these sequences were drawn randomly from the replay memory. More recently, Isele et al. (Isele and Cosgun, 2018) reported a selective experience replay approach aimed at performing well in the context of lifelong learning (Thrun, 1996). The authors of this work proposed a long term replay memory in addition to the conventionally used one. Certain bases for designing this long-term replay memory, such as favoring transitions associated with high rewards and high absolute TD errors are similar to the ones described in the present work. However, the approach does not explore the replay of sequences, and its fundamental purpose is to shield against catastrophic forgetting (Goodfellow et al., 2013) when multiple tasks are learned in sequence. The replay approach described in the present work focuses on enabling more sample-efficient learning in situations where positive rewards occur rarely. Apart from this, Andrychowicz et al. (2017) proposed a hindsight experience replay approach, directed at addressing this problem, where each episode is replayed with a goal that is different from the original goal of the agent. The authors reported significant improvements in the learning performance in problems with sparse and binary rewards. These improvements were essentially brought about by allowing the learned value/*Q* values (which would otherwise remain mostly unchanged due to the sparsity of rewards) to undergo significant change under the influence of an arbitrary goal. The underlying idea behind our approach also involves modification of the *Q*−values in reward-sparse regions of the state-action space. The modifications, however, are not based on arbitrary goals, and are selectively performed on state-action pairs associated with successful transition sequences associated with high absolute TD errors. Nevertheless, the hindsight replay approach is orthogonal to our proposed approach, and hence, could be used in conjunction with it.

Much like in Schaul et al. (2016), TD errors have been frequently used as a basis for prioritization in other RL problems (Thrun, 1992; White et al., 2014; Schaul et al., 2016). In particular, the model-based approach of prioritized sweeping (Moore and Atkeson, 1993; van Seijen and Sutton, 2013) prioritizes backups that are expected to result in a significant change in the value function.

The algorithm we propose here uses a model-free architecture, and it is based on the idea of selectively reusing previous experience. However, we describe the reuse of sequences of transitions based on the TD errors observed when these transitions take place. Replaying sequences of experiences also seems to be biologically plausible (Buhry et al., 2011; Ólafsdóttir et al., 2015). In addition, it is known that animals tend to remember experiences that lead to high rewards (Singer and Frank, 2009). This is an idea reflected in our work, as only those transition sequences that lead to high rewards are considered for being stored in the replay memory. In filtering transition sequences in this manner, we simultaneously address the issue of determining which experiences are to be stored.

In addition to selecting transition sequences, we also generate virtual sequences of transitions which the agent could have possibly experienced, but in reality, did not. This virtual experience is then replayed to improve the agent's learning. Some early approaches in RL, such as the dyna architecture (Sutton, 1990) also made use of simulated experience to improve the value function estimates. However, unlike the approach proposed here, the simulated experience was generated based on models of the reward function and transition probabilities which were continuously updated based on the agent's interactions with the environment. In this sense, the virtual experience generated in our approach is more grounded in reality, as it is based directly on the data collected through the agent-environment interaction. In more recent work, Fonteneau et al. describe an approach to generate artificial trajectories and use them to find policies with acceptable performance guarantees (Fonteneau et al., 2013). However, this approach is designed for batch RL, and the generated artificial trajectories are not constructed using a TD error basis. Our approach also recognizes the real-world limitations of replay memory (de Bruin et al., 2015), and stores only a certain amount of information at a time, specified by memory parameters. The selected and generated sequences are stored in the replay memory in the form of libraries which are continuously updated so that the agent is equipped with transition sequences that are most relevant to the task at hand.

**Figure 1:**
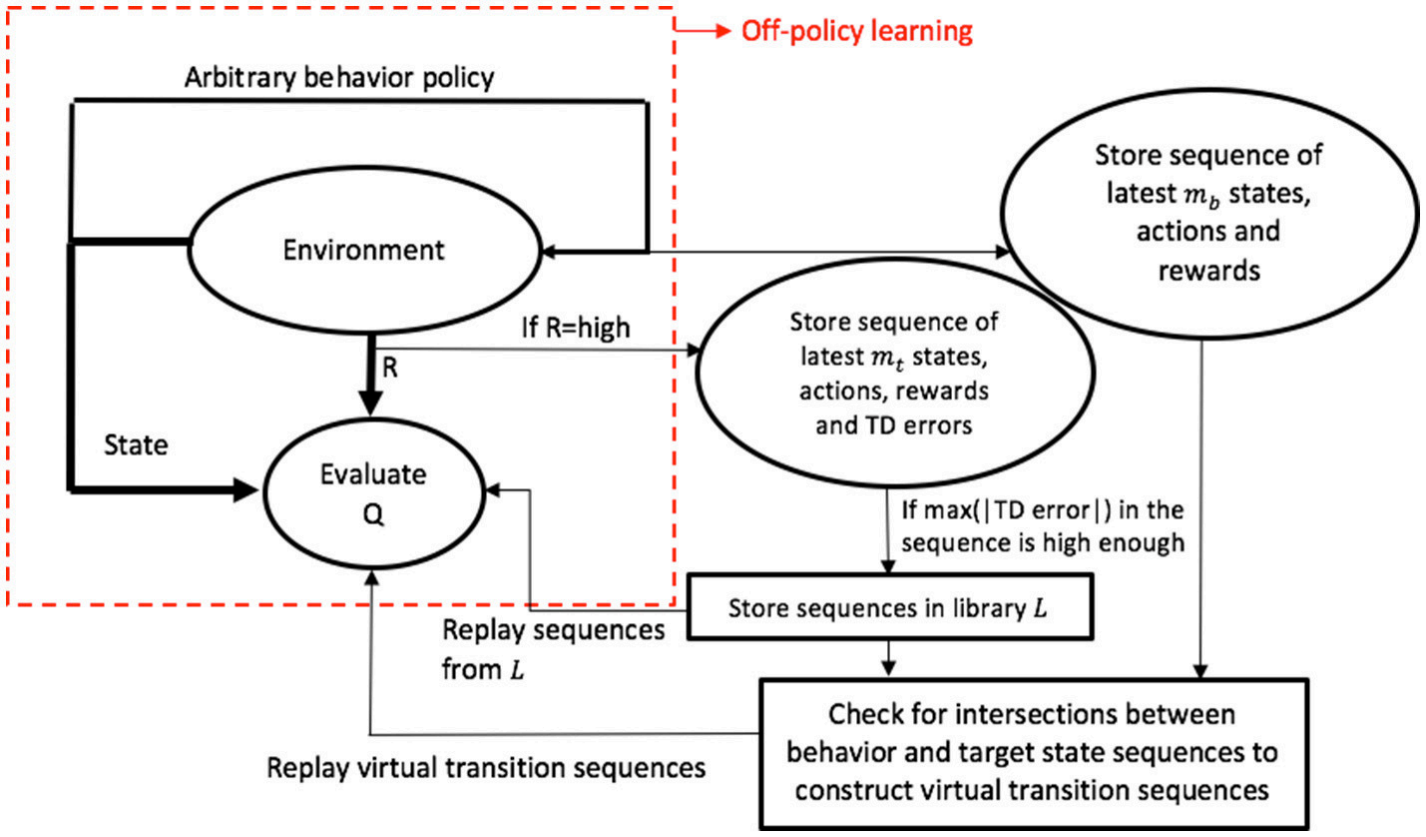
Structure of the proposed algorithm in contrast to the traditional off-policy structure. *Q* and *R* denote the action-value function and reward, respectively.

## 3. Methodology

The idea of selecting appropriate transition sequences for replay is relatively straightforward. In order to improve the agent's learning, first, we simply keep track of the state, actions, rewards, and absolute values of the TD errors associated with each transition. Generally, in difficult learning environments, high rewards occur rarely. So, when such an event is observed, we consider storing the corresponding sequence of transitions into a replay library *L*. In this manner, we use the reward information as a means to filter transition sequences. The approach is similar to that used by Narasimhan et al. (2015), where transitions associated with positive rewards are prioritized for replay.

Among the transition sequences considered for inclusion in the library *L*, those containing transitions with high absolute TD error values are considered to be the ones with high potential for learning progress. Hence, they are accordingly prioritized for replay. The key idea is that when the TD error associated with a particular transition is large in magnitude, it generally implies a proportionately greater change in the value of the corresponding state/state-action pair. Such large changes have the potential to influence the values of the states/state-action pairs leading to it, which implies a high potential for learning. Hence, prioritizing such sequences of transitions for replay is likely to bring about greater learning progress. Transition sequences associated with large magnitudes of TD error are retained in the library, while those with lower magnitudes are removed and replaced with superior alternatives. In reality, such transition sequences may be very long and hence, impractical to store. Due to such practical considerations, we store only a portion of the sequence, based on a predetermined memory parameter. The library is continuously updated as and when the agent-environment interaction takes place, such that it will eventually contain sequences associated with the highest absolute TD errors.

As described earlier, replaying suitable sequences allows the effects of large changes in value functions to be propagated throughout the sequence. In order to propagate this information even further to other regions of the state/state-action space, we use the sequences in *L* to construct additional transition sequences which could have possibly occurred. These virtual sequences are stored in another library *L*_*v*_, and later used for experience replay.

**Figure 2:**
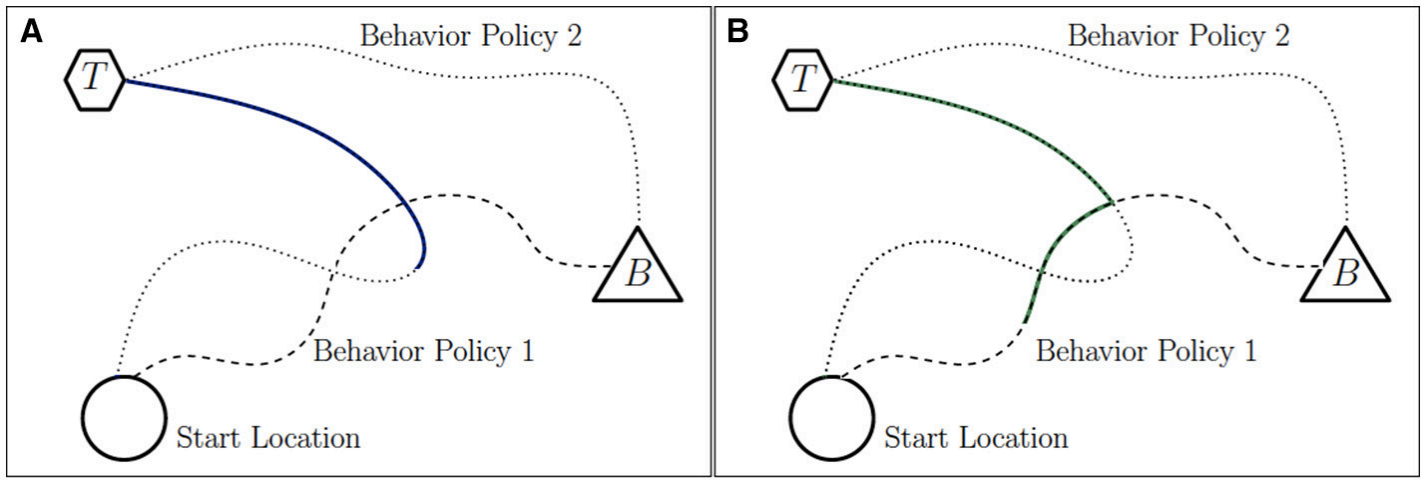
**(A)** Trajectories corresponding to two hypothetical behavior policies are shown. A portion of the trajectory associated with a high reward (and stored in *L*) is highlighted. **(B)** The virtual trajectory constructed from the two behavior policies is highlighted. The states, actions, and rewards associated with this trajectory constitute a virtual transition sequence.

In order to intuitively describe our approach of artificially constructing sequences, we consider the hypothetical example shown in Figure 2A, where an agent executes behavior policies that help it learn to navigate toward location *B* from the start location. However, using off-policy learning, we aim to learn value functions corresponding to the policy that helps the agent navigate toward location *T*.

The trajectories shown in Figure 2A correspond to hypothetical actions dictated by the behavior policy midway through the learning process, during two separate episodes. The trajectories begin at the start location and terminate at location *B*. However, the trajectory corresponding to behavior policy 2 also happens to pass through location *T*, at which point the agent receives a high reward. This triggers the transition sequence storage mechanism described earlier, and we assume that some portion of the sequence (shown by the highlighted portion of the trajectory in Figure 2A) is stored in library *L*. Behavior policy 1 takes the agent directly from the start location toward the location *B*, where it terminates. As the agent moves along its trajectory, it intersects with the state trajectory corresponding to the sequence stored in *L*. Using this intersection, it is possible to artificially construct additional trajectories (and their associated transition sequences) that are successful with respect to the task of navigating to location *T*. The highlighted portions of the trajectories corresponding to the two behavior policies in Figure 2B show such a state trajectory, constructed using information related to the intersection of portions of the two previously observed trajectories. The state, action, and reward sequences associated with this highlighted trajectory form a virtual transition sequence.

Such artificially constructed transition sequences present the possibility of considerable learning progress. This is because, when replayed, they help propagate the large learning potential (characterized by large magnitudes of TD errors) associated with sequences in *L* to other regions of the state/state-action space. These replay updates supplement the off-policy value function updates that are carried out in parallel, thus accelerating the learning of the task in question. This outlines the basic idea behind our approach.

Fundamentally, our approach can be decomposed into three steps:

1. Tracking and storage of relevant transition sequences
2. Construction of virtual transition sequences using the stored transition sequences
3. Replaying the transition sequences

These steps are explained in detail in sections 3.1, 3.2, and 3.3.

### 3.1. Tracking and storage of relevant transition sequences

As described, virtual transition sequences are constructed by joining together two transition sequences. One of them, say Θ_*t*_, composed of *m*_*t*_ transitions, is historically successful—it has experienced high rewards with respect to the task, and is part of the library *L*. The other sequence, Θ_*b*_, is simply a sequence of the latest *m*_*b*_ transitions executed by the agent.

If the agent starts at state *s*_0_ and moves through intermediate states *s*_*i*_ and eventually to *s*_*j*+1_ (most recent state) by executing a series of actions *a*_0_…*a*_*i*_…*a*_*j*_, it receives rewards *R*_0_…*R*_*i*_…*R*_*j*_ from the environment. These transitions comprise the transition sequence Θ_*b*_.

(1) $$\Theta_{b}=\{\begin{array}{l} {}[S(0:j)\;\pi (0:j)\;R(0:j)]\;\;\;\;\;\;\text{if }j\leq m_{b}\;\\ {}[S((j-m_{b}):j)\;\pi ((j-m_{b}):j)\;R((j-m_{b}):j)]\text{ otherwise } \end{array}$$

where:

$$\begin{array}{l} S\left(x:y\right)=\left(s_{x}\ldots s_{i}\ldots s_{y}\right),\\ \pi \left(x:y\right)=\left(a_{x}\ldots a_{i}\ldots a_{y}\right),\\ R\left(x:y\right)=\left(R_{x}\ldots R_{i}\ldots R_{y}\right). \end{array}$$

We respectively refer to *S*(*x* : *y*), π(*x* : *y*), and *R*(*x* : *y*) as the state, action, and reward transition sequences corresponding to

(2) $$\Theta_{t}=\{\begin{array}{l} {}[S(0:k)\;\pi (0:k)\;R(0:k)\;\Delta (0:k)]\;\;\;\;\;\;\text{if }k\leq m_{t}\;\\ {}[S((k-m_{t}):k)\;\pi ((k-m_{t}):k)\;R((k-m_{t}):k)\;\Delta ((k-m_{t}):k)]\text{ otherwise } \end{array}$$

a series of agent-environment interactions, indexed from *x* to *y* (*x, y*∈ ℕ).

For the case of the transition sequence Θ_*t*_, we keep track of the sequence of TD errors δ_0_…δ_*i*_…δ_*k*_ observed as well. If a high reward is observed in transition *k*, then:

where Δ(*x* : *y*) = (|δ_*x*_|…|δ_*i*_|…|δ_*y*_|).

The memory parameters *m*_*b*_ and *m*_*t*_ are chosen based on the memory constraints of the agent. They determine how much of the recent agent-environment interaction history is to be stored in memory.

It is possible that the agent encounters a number of transitions associated with high rewards while executing the behavior policy. Corresponding to these transitions, a number of successful transition sequences Θ_*t*_ would also exist. These sequences are maintained in the library *L* in a manner similar to the *Policy Library through Policy Reuse (PLPR)* algorithm (Fernández and Veloso, 2005). To decide whether to include a new transition sequence Θ_*t*_new__ into the library *L*, we determine the maximum value of the absolute TD error sequence Δ corresponding to Θ_*t*_new__ and check whether it is τ-close—the parameter τ determines the exclusivity of the library—to the maximum of the corresponding values associated with the transition sequences in *L*. If this is the case, then Θ_*t*_new__ is included in *L*. Since the transition sequences are filtered based on the maximum of the absolute values of TD errors among all the transitions in a sequence, this approach should be able to mitigate problems stemming from low magnitudes of TD errors associated with local optima (Baird, 1999; Tutsoy and Brown, 2016b). Using the absolute TD error as a basis for selection, we maintain a fixed number (*l*) of transition sequences in the library *L*. This ensures that the library is continuously updated with the latest transition sequences associated with the highest absolute TD errors. The complete algorithm is illustrated in Algorithm 1.

**Algorithm 1:** Maintaining a replay library of transition sequences

1: **Inputs:**
τ: Parameter that determines the exclusivity of the library
*l*: Parameter that determines the number of transition sequences allowed in the library
Δ_*k*_: Sequence of absolute TD errors corresponding to a transition sequence Θ_*k*_
*L* = {Θ_*t*0_…Θ_*t*_*i*__…Θ_*t*_*m*__}: A library of transition sequences (*m* ≤ *l*)
Θ_*t*_new__: New transition sequence to be evaluated
2: *W*_new_ = max(Δ_*t*_new__)
3: **for** *j* = 1:*m* **do**
4: *W*_*j*_ = max(Δ_*t*_*j*__)
5: **end for**
6: **if** *W*_new_ * τ > max(*W*) **then**
7: *L* = *L*∪{Θ_*t*_new__}
8: *n*_*t*_ = Number of transition sequences in *L*
9: **if** *n*_*t*_ > *l* **then**
10: *L* = {Θ_*t*_*n*_*t*__−*l*_…Θ_*t*_*i*__…Θ_*t*_*n*_*t*___}
11: **end if**
12: **end if**

### 3.2. Virtual transition sequences

Once the transition sequence Θ_*b*_ is available and a library *L* of successful transition sequences Θ_*t*_ is obtained, we use this information to construct a library *L*_*v*_ of virtual transition sequences Θ_*v*_. The virtual transition sequences are constructed by first finding points of intersection *s*_*c*_ in the state transition sequences of Θ_*b*_ and the Θ_*t*_'s in *L*.

Let us consider the transition sequence Θ_*b*_:

$$\begin{array}{l} \Theta_{b}=\left[S\left(x:y\right)\;\pi \left(x:y\right)\;R\left(x:y\right)\right], \end{array}$$

and a transition sequence Θ_*t*_:

$$\begin{array}{l} \Theta_{t}=\left[S\left(x^{\prime }:y^{\prime }\right)\;\pi \left(x^{\prime }:y^{\prime }\right)\;R\left(x^{\prime }:y^{\prime }\right)\;\Delta \left(x^{\prime }:y^{\prime }\right)\right], \end{array}$$

Let Θ_*t*_*s*__ be a sub-matrix of Θ_*t*_ such that:

(3) $$\begin{array}{r} \Theta_{t_{s}}=\left[S\left(x^{\prime }:y^{\prime }\right)\;\pi \left(x^{\prime }:y^{\prime }\right)\;R\left(x^{\prime }:y^{\prime }\right)\right], \end{array}$$

Now, if $\sigma_{x}^{y}$ and $\sigma_{x^{\prime }}^{y^{\prime }}$ are sets containing all the elements of sequences *S*(*x* : *y*) and *S*(*x*′ : *y*′), respectively, and if $\exists s_{c}\in \left\{\sigma_{x}^{y}\cap \sigma_{x^{\prime }}^{y^{\prime }}\right\}$, then:

$$\begin{array}{l} S\left(x:y\right)=\left(s_{x},\ldots s_{c},s_{c+1},\ldots s_{y}\right), \end{array}$$

and

$$\begin{array}{l} S\left(x^{\prime }:y^{\prime }\right)=\left(s_{x^{\prime }}\ldots s_{c},s_{c+1}\ldots s_{y^{\prime }}\right). \end{array}$$

Once points of intersection have been obtained as described above, each of the two sequences Θ_*b*_ and Θ_*t*_*s*__ are decomposed into two subsequences at the point of intersection such that:

(4) $$\Theta_{b}=\left[\begin{array}{c} \Theta_{b}^{1}\\ \Theta_{b}^{2} \end{array}\right]$$

where $\Theta_{b}^{1}=\left[S\left(x:c\right)\;\pi \left(x:c\right)\;R\left(x:c\right)\right]$

and $\Theta_{b}^{2}=\left[S\left(\left(c+1\right):y\right)\;\pi \left(\left(c+1\right):y\right)\;R\left(\left(c+1\right):y\right)\right]$

Similarly,

(5) $$\Theta_{t_{s}}=\left[\begin{array}{c} \Theta_{t_{s}}^{1}\\ \Theta_{t_{s}}^{2} \end{array}\right]$$

where

$\Theta_{t_{s}}^{1}=\left[S\left(x^{\prime }:c\right)\;\pi \left(x^{\prime }:c\right)\;R\left(x^{\prime }:c\right)\right]$

and

$\Theta_{t_{s}}^{2}=\left[S\left(\left(c+1\right):y^{\prime }\right)\;\pi \left(\left(c+1\right):y^{\prime }\right)\;R\left(\left(c+1\right):y^{\prime }\right)\right]$

The virtual transition sequence is then simply:

(6) $$\begin{array}{r} \Theta_{v}=\left[\begin{array}{c} \Theta_{b}^{1}\\ \Theta_{t_{s}}^{2} \end{array}\right] \end{array}$$

We perform the above procedure for each transition sequence in *L* to obtain the corresponding virtual transition sequences Θ_*v*_. These virtual transition sequences are stored in a library *L*_*v*_:

$$\begin{array}{l} L_{v}=\left\{{\Theta_{v}}_{1}\ldots {\Theta_{v}}_{i}\ldots {\Theta_{v}}_{n_{v}}\right\}, \end{array}$$

where *n*_*v*_ denotes the number of virtual transition sequences in *L*_*v*_, subjected to the constraint *n*_*v*_ ≤ *l*.

The overall process for constructing and storing virtual transition sequences is summarized in Algorithm 2. Once the library *L*_*v*_ has been constructed, we replay the sequences contained in it to improve the estimates of the value function. The details of this are discussed in section 3.3.

**Algorithm 2:** Constructing virtual transition sequences

1: **Inputs:**
Sequence of latest *m*_*b*_ transitions Θ_*b*_
Library *L* containing *n*_*t*_ stored transition sequences
Library *L*_*v*_ for storing virtual transition sequences
2: **for** *t* = 1:*n*_*t*_ **do**
3: Extract Θ_*t*_*s*__ from Θ_*t*_ (Equation 3)
4: Find set of states *S*_*I*_ corresponding to the intersection of the state trajectories of Θ_*b*_ and Θ_*t*_*s*__
5: **if** *S*_*I*_ is not empty, **then**
6: **for** each state *s*_*i*_ in *S*_*I*_, **do**
7: Treat *s*_*i*_ as the intersection point and decompose Θ_*b*_ and Θ_*t*_*s*__ as per Equations 4 and 5
8: **end for**
9: Choose *s*_*c*_ from *S*_*I*_ such that the number of transitions in $\Theta_{b}^{1}$ is maximized
10: **end if**
11: Use the selected *s*_*c*_ to construct the virtual transition sequence Θ_*v*_ as per Equation 6
12: Use library *L*_*v*_ to store the constructed sequence (*L*_*v*_ = *L*_*v*_∪{Θ_*v*_})
13: **end for**

### 3.3. Replaying the transition sequences

In order to make use of the transition sequences described, each of the state-action-reward triads {*s a r*} in the transition sequence *L*_*v*_ is replayed as if the agent had actually experienced them.

Similarly, sequences in *L* are also be replayed from time to time. Replaying sequences from *L* and *L*_*v*_ in this manner causes the effects of large absolute TD errors originating from further up in the sequence to propagate through the respective transitions, ultimately leading to more accurate estimates of the value function. The transitions are replayed as per the standard *Q*-learning update equation shown below:

(7) $$\begin{array}{r} Q\left(s_{j},a_{j}\right)\leftarrow Q\left(s_{j},a_{j}\right)+\alpha \left[R\left(s_{j},a_{j}\right)+\gamma \underset{a^{\prime }}{\max}Q\left(s_{j+1},a^{\prime }\right)-Q\left(s_{j},a_{j}\right)\right]. \end{array}$$

Where *s*_*j*_ and *a*_*j*_ refer to the state and action at transition *j*, and *Q* and *R* represent the action-value function and reward corresponding to the task. The variable *a*′ is a bound variable that represents any action in the action set A. The learning rate and discount parameters are represented by α and γ respectively.

The sequence Θ_*t*_*s*__ in Equation (6) is a subset of Θ_*t*_, which is in turn part of the library *L* and thus associated with a high absolute TD error. When replaying Θ_*v*_, the effects of the high absolute TD errors propagate from the values of state/state-action pairs in $\Theta_{t_{s}}^{2}$ to those in $\Theta_{b}^{1}$. Hence, in case of multiple points of intersection, we consider points that are furthest down Θ_*b*_. In other words, the intersection point is chosen to maximize the length of $\Theta_{b}^{1}$. In this manner, a larger number of state-action values experience improvements brought about by replaying the transition sequences.

**Algorithm 3:** Replay of virtual transition sequences from library *L*_*v*_

1: **Inputs:**
α: learning rate
γ: discount factor
*L*_*v*_ = {Θ_*v*_0__…Θ_*v*_*i*__…Θ_*v*_*n*_*v*___}: A library of virtual transition sequences with *n*_*v*_ sequences
2: **for** *i* = 1:*n*_*v*_ **do**
3: *n*_sar_ = number of {*s a r*} triads in Θ_*v*_*i*__
4: *j* = 1
5: **while** *j* ≤ *n*_*sar*_ **do**
6: $Q\left(s_{j},a_{j}\right)\leftarrow Q\left(s_{j},a_{j}\right)+\alpha \left[R\left(s_{j},a_{j}\right)+\gamma \max_{a^{\prime }}Q\left(s_{j+1},a^{\prime }\right)-Q\left(s_{j},a_{j}\right)\right]$
7: *j* ← *j*+1
8: **end while**
9: **end for**

## 4. Results and discussion

**Figure 3:**
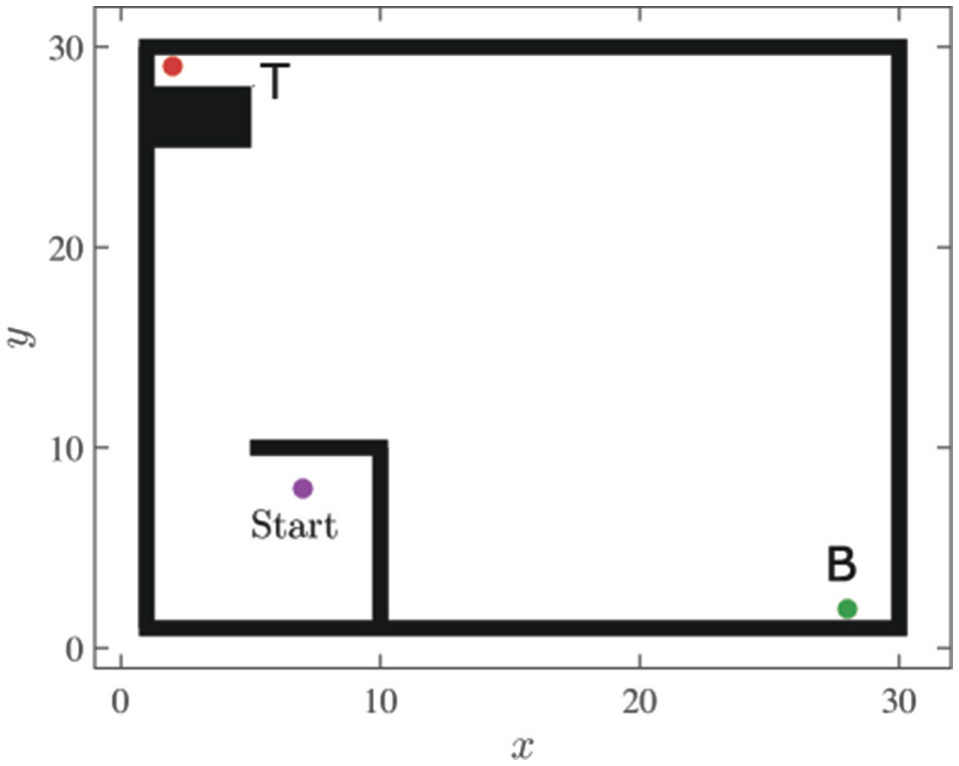
Navigation environment used to demonstrate the approach of replaying transition sequences.

We demonstrate our approach on modified versions of two standard reinforcement learning tasks. The first is a multi-task navigation/puddle-world problem (Figure 3), and the second is a multi-task mountain car problem (**Figure 6**). In both these problems, behavior policies are generated to solve a given task (which we refer to as the primary task) relatively greedily, while the value function for another task of interest (which we refer to as the secondary task) is simultaneously learned in an off-policy manner. The secondary task is intentionally made more difficult by making appropriate modifications to the environment. Such adverse multi-task settings best demonstrate the effectiveness of our approach and emphasize its advantages over other experience replay approaches. We characterize the difficulty of the secondary task with a difficulty ratio ρ, which is the fraction of the executed behavior policies that experience a high reward with respect to the secondary task. A low value of ρ indicates that achieving the secondary task under the given behavior policy is difficult. In both tasks, the *Q*− values are initialized with random values, and once the agent encounters the goal state of the primary task, the episode terminates.

### 4.1. Navigation/puddle-world task

In the navigation environment, the simulated agent is assigned tasks of navigating to certain locations in its environment. We consider two locations, *B* and *T*, which represent the primary and secondary task locations respectively. The environment is set up such that the location corresponding to high rewards with respect to the secondary task lies far away from that of the primary task (see Figure 3). In addition to this, the accessibility to the secondary task location is deliberately limited by surrounding it with obstacles on all but one side. These modifications contribute toward a low value of ρ, especially when the agent operates with a greedy behavior policy with respect to the primary task.

The agent is assumed to be able to sense its location in the environment accurately, and can detect when it “bumps” into an obstacle. It can move around in the environment at a maximum speed of 1 unit per time step by executing actions to take it forwards, backwards, sideways, and diagonally forwards or backwards to either side. In addition to these actions, the agent can choose to hold its current position. However, the transitions resulting from these actions are probabilistic in nature. The intended movements occur only 80% of the time, and for the remaining 20%, the *x*- and *y*-coordinates may deviate from their intended values by 1 unit. Also, the agent's location does not change if the chosen action forces it to run into an obstacle.

The agent employs *Q*-learning with a relatively greedy policy (ϵ = 0.1) that attempts to maximize the expected sum of primary rewards. The reward structure for both tasks is such that the agent receives a high reward (100) for visiting the respective goal locations, and a high penalty (−100) for bumping into an obstacle in the environment. In addition to this, the agent is assigned a living penalty (−10) for each action that fails to result in the goal state. In all simulations, the discount factor γ is set to be 0.9, the learning rate α is set to a value of 0.3 and the parameter τ mentioned in Algorithm 1 is set to be 1. Although various approaches exist to optimize the values of the *Q*−learning hyperparameters (Garcia and Ndiaye, 1998; Even-Dar and Mansour, 2003; Tutsoy and Brown, 2016a), the values were chosen arbitrarily, such that satisfactory performances were obtained for both the navigation as well as the mountain-car environments.

**Figure 4:**
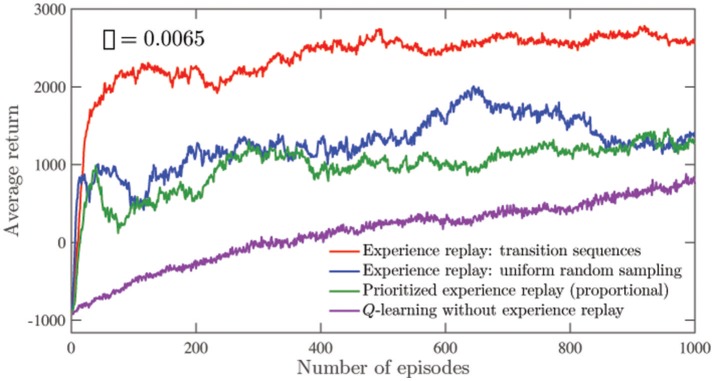
Comparison of the average secondary returns over 50 runs using different experience replay approaches as well as *Q*-learning without experience replay in the navigation environment. The standard errors are all <300. For the different experience replay approaches, the number of replay updates are controlled to be the same.

In the environment described, the agent executes actions to learn the primary task. Simultaneously, the approach described in section 3 is employed to learn the value functions associated with the secondary task. At each episode of the learning process, the agent's performance with respect to the secondary task is evaluated. In order to compute the average return for an episode, we allow the agent to execute *n*_*ga*_(= 100) greedy actions from a randomly chosen starting point, and record the accumulated reward. The process is repeated for *n*_*trials*_(= 100) trials, and the average return for the episode is reported as the average accumulated reward per trial. The average return corresponding to each episode in Figure 4 is computed in this way. The mean of these average returns over all the episodes is reported as *G*_*e*_ in Table 1. That is, the average return corresponding to the *k*^*th*^episode *g*_*k*_ is given by:

$$g_{k}=\frac{\overset{n_{trials}}{\underset{i\;=\;1}{\sum }}\overset{n_{ga}}{\underset{j\;=\;1}{\sum }}R_{ij}}{n_{trials}}$$

and

$$G_{e}=\frac{\overset{N_{E}}{\underset{k\;=\;1}{\sum }}g_{k}}{N_{E}}$$

Where *R*_*ij*_ is the reward obtained by the agent in a step corresponding to the greedy action *j*, in trial *i*, and *N*_*E*_ is the maximum number of episodes.

**Table 1:** Average secondary returns accumulated per episode (*G*_*e*_) using different values of the memory parameters in the navigation environment.

**A**	
*m*_*b*_	*G*_*e*_
10	1559.7
100	2509.7
1,000	2610.4
**B**	
*m*_*t*_	*G*_*e*_
10	1072.5
100	1159.2
1,000	2610.4
**C**	
*n*_*v*_	*G*_*e*_
10	2236.6
50	2610.4
100	2679.5

With regular Q-learning (without experience replay), G_e_ = 122.9

Figure 4 shows the average return for the secondary task plotted for 50 runs of 1,000 learning episodes using different learning approaches. The low average value of ρ (= 0.0065 as indicated in Figure 4) indicates the relatively high difficulty of the secondary task under the behavior policy being executed. As observed in Figure 4, an agent that replays transition sequences manages to accumulate high average returns at a much faster rate as compared to regular *Q*-learning. The approach also performs better than other experience replay approaches for the same number of replay updates. These replay approaches are applied independently of each other for the secondary task. In Figure 4, the prioritization exponent for prioritized experience replay is set to 1.

Table 1 shows the average return for the secondary task accumulated per episode (*G*_*e*_) during 50 runs of the navigation task for different values of memory parameters *m*_*b*_, *m*_*t*_ and *n*_*v*_ used in our approach. Each of the parameters are varied separately while keeping the other parameters fixed to their default values. The default values used for *m*_*b*_, *m*_*t*_, and *n*_*v*_ are 1,000, 1,000, and 50, respectively.

#### Application to the primary task

**Figure 5:**
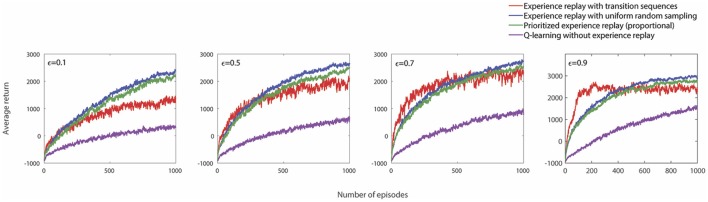
The performance of different experience replay approaches on the primary task in the navigation environment for different values of the exploration parameter ϵ, averaged over 30 runs. For these results, the memory parameters used are as follows: *m*_*b*_ = 1, 000, *m*_*t*_ = 1, 000, and *n*_*v*_ = 50.

In the simulations described thus far, the performance of our approach was evaluated on a secondary task, while the agent executed actions relatively greedily with respect to a primary task. Such a setup was chosen in order to ensure a greater sparsity of high rewards for the secondary task. However, the proposed approach of replaying sequences of transitions can also be applied to the primary task in question. In particular, when a less greedy exploration strategy is employed (that is, when ϵ is high), such conditions of reward-sparsity can be recreated for the primary task. Figure 5 shows the performance of different experience replay approaches when applied to the primary task, for different values of ϵ. As expected, for more exploratory behavior policies, which correspond to lower probabilities of obtaining high rewards, the approach of replaying transition sequences is significantly beneficial, especially at the early stages of learning. However, as the episodes progress, the effects of drastically large absolute TD errors would have already penetrated into other regions of the state-action space, and the agent ceases to benefit as much from replaying transition sequences. Hence, other forms of replay such as experience replay with uniform random sampling, or prioritized experience replay were found to be more useful after the initial learning episodes.

### 4.2. Mountain car task

**Figure 6:**
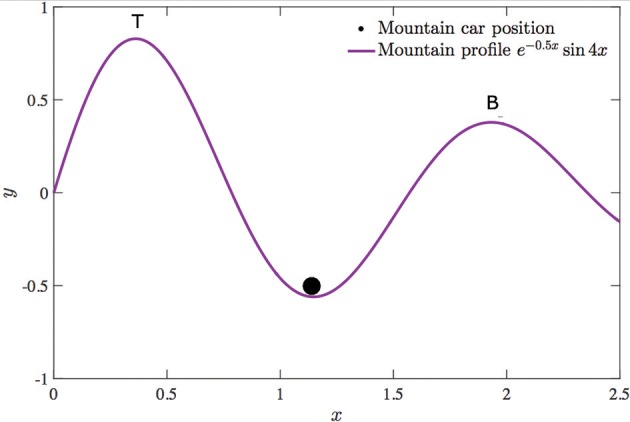
Mountain car environment used to demonstrate off-policy learning using virtual transition sequences.

In the mountain car task, the agent, an under-powered vehicle represented by the circle in Figure 6 is assigned a primary task of getting out of the trough and visiting point *B*. The act of visiting point *T* is treated as the secondary task. The agent is assigned a high reward (100) for for fulfilling the respective objectives, and a living penalty (−1) is assigned for all other situations. At each time step, the agent can choose from three possible actions: (1) accelerating in the positive *x* direction, (2) accelerating in the negative *x* direction, and (3) applying no control. The environment is discretized such that 120 unique positions and 100 unique velocity values are possible.

The mountain profile is described by the equation *y* = *e*^−0.5*x*^ sin(4*x*) such that point *T* is higher than *B*. Also, the average slope leading to *T* is steeper than that leading to *B*. In addition to this, the agent is set to be relatively greedy with respect to the primary task, with an exploration parameter ϵ = 0.1. These factors make the secondary task more difficult, resulting in a low value of ρ (= 0.0354) under the policy executed.

**Figure 7:**
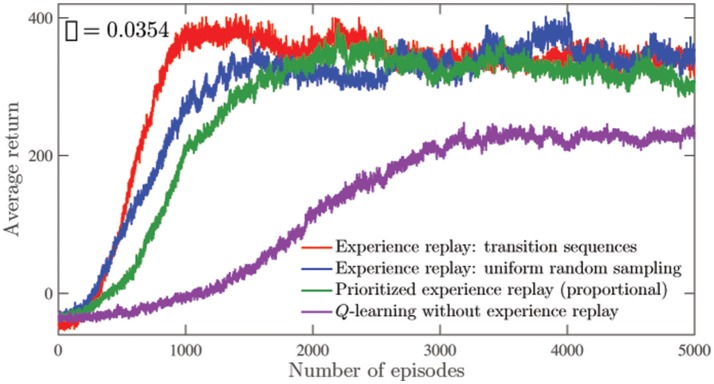
Comparison of the average secondary returns over 50 runs using different experience replay approaches as well as *Q*-learning without experience replay in the mountain-car environment. The standard errors are all <85. For the different experience replay approaches, the number of replay updates are controlled to be the same.

Figure 7 shows the average secondary task returns for 50 runs of 5, 000 learning episodes. It is seen that especially during the initial phase of learning, the agent accumulates rewards at a higher rate as compared to other learning approaches. As in the navigation task, the number of replay updates are restricted to be the same while comparing the different experience replay approaches in Figure 7. Analogous to Table 1, Table 2 shows the average secondary returns accumulated per episode (*G*_*e*_) over 50 runs in the mountain-car environment, for different values of the memory parameters. The default values for *m*_*b*_, *m*_*t*_, and *n*_*v*_ are the same as those mentioned in the navigation environment, that is, 1,000, 1,000, and 50, respectively.

**Table 2:** Average secondary returns accumulated per episode (*G*_*e*_) using different values of the memory parameters in the mountain car environment.

**A**	
*m*_*b*_	*G*_*e*_
10	221.0
100	225.1
1,000	229.9
**B**	
*m*_*t*_	*G*_*e*_
10	129.9
100	190.5
1,000	229.9
**C**	
*n*_*v*_	*G*_*e*_
10	225.6
50	229.9
100	228.4

With regular Q-learning (without experience replay), G_e_ = 132.9

From Figures 4, 7, the agent is seen to be able to accumulate significantly higher average secondary returns per episode when experiences are replayed. Among the experience replay approaches, the approach of replaying transition sequences is superior for the same number of replay updates. This is especially true in the navigation environment, where visits to regions associated with high secondary task rewards are much rarer, as indicated by the low value of ρ. In the mountain car problem, the visits are more frequent, and the differences between the different experience replay approaches are less significant. The value of the prioritization exponent used here is the same as that used in the navigation task. The approach of replaying sequences of transitions also offers noticeable performance improvements when applied to the primary task (as seen in Figure 5), especially during the early stages of learning, and when highly exploratory behavior policies are used. In both the navigation and mountain-car environments, the performances of the approaches that replay individual transitions—experience replay with uniform random sampling and prioritized experience replay—are found to be nearly equivalent. We have not observed a significant advantage of using the prioritized approach, as reported in previous studies (Schaul et al., 2016; Hessel et al., 2017) using deep RL. This perhaps indicates that improvements brought about by the prioritized approach are much more pronounced in deep RL applications.

The approach of replaying transition sequences seems to be particularly sensitive to the memory parameter *m*_*t*_, with higher average returns being achieved for larger values of *m*_*t*_. A possible explanation for this could simply be that larger values of *m*_*t*_ correspond to longer Θ_*t*_ sequences, which allow a larger number of replay updates to occur in more regions of the state/state-action space. The influence of the length of the Θ_*b*_ sequence, specified by the parameter *m*_*b*_ is also similar in nature, but its impact on the performance is less emphatic. This could be because longer Θ_*b*_ sequences allow a greater chance for their state trajectories to intersect with those of Θ_*t*_, thus improving the chances of virtual transition sequences being discovered, and of the agent's value functions being updated using virtual experiences. However, the parameter *n*_*v*_, associated with the size of the library *L*_*v*_ does not seem to have a noticeable influence on the performance of this approach. This is probably due to the fact that the library *L* (and consequently *L*_*v*_) is continuously updated with new, suitable transition sequences (successful sequences associated with higher magnitudes of TD errors) as and when they are observed. Hence, the storage of a number of transition sequences in the libraries becomes largely redundant.

Although the method of constructing virtual transition sequences is more naturally applicable to the tabular case, it could also possibly be extended to approaches with linear and non-linear function approximation. However, soft intersections between state trajectories would have to be considered instead of absolute intersections. That is, while comparing the state trajectories *S*(*x* : *y*) and *S*(*x*′:*y*′), the existence of *s*_*c*_ could be considered if it is close to elements in both *S*(*x* : *y*) and *S*(*x*′ : *y*′) within some specified tolerance limit. Such modifications could allow the approach described here to be applied to deep RL. Transitions that belong to the sequences Θ_*v*_ and Θ_*t*_ could then be selectively replayed, thereby bringing about improvements in the sample efficiency. However, the experience replay approaches (implemented with the mentioned modifications) applied to the environments described in section 4 did not seem to bring about significant performance improvements when a neural network function approximator was used. The performance of the corresponding deep Q-network (DQN) was approximately the same even without any experience replay. This perhaps, reveals that the performance of the proposed approach needs to be evaluated on more complex problems such as the Atari domain (Mnih et al., 2015). Reliably implementing virtual transition sequences to the function approximation case could be a future area of research. One of the limitations of constructing virtual transition sequences is that in higher dimensional spaces, intersections in the state trajectories become less frequent, in general. However, other sequences in the library *L* can still be replayed. If appropriate sequences have not yet been discovered or constructed, and are thus not available for replay, other experience replay approaches that replay individual transitions can be used to accelerate learning in the meanwhile.

Perhaps another limitation of the approach described here is that constructing the library *L* requires some notion of a goal state associated with high rewards. By tracking the statistical properties such as the mean and variance of the rewards experienced by an agent in its environment in an online manner, the notion of what qualifies as a high reward could be automated using suitable thresholds (Karimpanal and Wilhelm, 2017). In addition to this, other criteria such as the returns or average absolute TD errors of a sequence could also be used to maintain the library.

**Figure 8:**
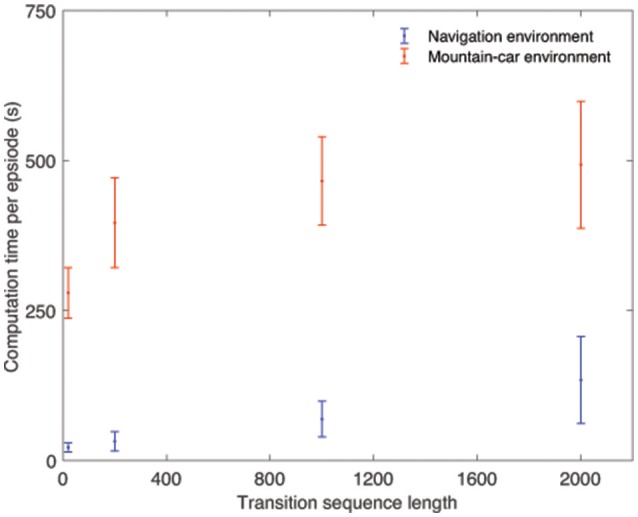
The variation of computational time per episode with sequence length for the two environments, computed over 30 runs.

It is worth adding that the memory parameters *m*_*b*_, *m*_*t*_, and *n*_*v*_ have been set arbitrarily in the examples described here. Selecting appropriate values for these parameters as the agent interacts with its environment could be a topic for further research. Figure 8 shows the mean and standard deviations of the computation time per episode for different sequence lengths, over 30 runs. The figure suggests that the computation time increases as longer transition sequences are used, and the trend can be approximated to be linear. These results could also be used to inform the choice of values for *m*_*b*_ and *m*_*t*_ for a given application. The values shown in Figure 8 were obtained from running simulations on a computer with an Intel i7 processor running at 2.7 GHz, using 8 GB of RAM, running a Windows 7 operating system.

The approach of replaying transition sequences has direct applications in multi-task RL, where agents are required to learn multiple tasks in parallel. Certain tasks could be associated with the occurrence of relatively rare events when the agent operates under specific behavior policies. The replay of virtual transition sequences could further improve the learning in such tasks. Such as robotics, where exploration of the state/state-action space is typically expensive in terms of time and energy. By reusing the agent-environment interactions in the manner described here, reasonable estimates of the value functions corresponding to multiple tasks can be maintained, thereby improving the efficiency of exploration.

## 5. Conclusion

In this work, we described an approach to replay sequences of transitions to accelerate the learning of tasks in an off-policy setting. Suitable transition sequences are selected and stored in a replay library based on the magnitudes of the TD errors associated with them. Using these sequences, we showed that it is possible to construct virtual experiences in the form of virtual transition sequences, which could be replayed to improve an agent's learning, especially in environments where desirable events occur rarely. We demonstrated the benefits of this approach by applying it to versions of standard reinforcement learning tasks such as the puddle-world and mountain-car tasks, where the behavior policy was deliberately made drastically different from the target policy. In both tasks, a significant improvement in learning speed was observed compared to regular *Q*-learning as well as other forms of experience replay. Further, the influence of the different memory parameters used was described and evaluated empirically, and possible extensions to this work were briefly discussed. Characterized by controllable memory parameters and the potential to significantly improve the efficiency of exploration at the expense of some increase in computation, the approach of using replaying transition sequences could be especially useful in fields such as robotics, where these factors are of prime importance. The extension of this approach to the cases of linear and non-linear function approximation could find significant utility, and is currently being explored.

## Author contributions

TK conceived the idea, coded the simulations, performed the experiments, authored the manuscript. RB reviewed and edited the manuscript, provided advice regarding the presentation of some of the ideas, prepared some of the figures and authored some parts of the manuscript.

### Conflict of interest statement

The authors declare that the research was conducted in the absence of any commercial or financial relationships that could be construed as a potential conflict of interest.
